# SARS-CoV-2 Infection and Vaccination, Immune Dysregulation, and Cancer

**DOI:** 10.3390/vaccines14030255

**Published:** 2026-03-11

**Authors:** Dace Pjanova, Aysha Rafeeque

**Affiliations:** Institute of Microbiology and Virology, Riga Stradins University, LV-1067 Riga, Latvia; aysha.rafeeque@rsu.lv

**Keywords:** SARS-CoV-2, COVID-19, vaccination, immune dysregulation, chronic inflammation, cancer

## Abstract

Severe Acute Respiratory Syndrome Coronavirus 2 (SARS-CoV-2) infection induces heterogeneous immune responses that influence both acute disease severity and long-term immune remodeling. A key question in the context of infection and vaccination is whether SARS-CoV-2 exerts direct oncogenic effects or instead acts as a transient immunological stressor capable of reinforcing tumor-permissive pathways. Current evidence does not support classical viral oncogenesis. Rather, severe infection is characterized by early interferon (IFN) imbalance followed by NF-κB-dominant inflammatory amplification, promoting sustained IL-6/JAK–STAT3 and MAPK signaling, chronic cytokine production, metabolic reprogramming, and impaired antitumor immune surveillance. At the molecular level, viral structural proteins modulate host signaling networks. The spike (S1) protein engages TLR2/TLR4–MyD88 pathways, activating NF-κB and MAPK cascades, while the membrane (M) protein reinforces NF-κB–STAT3 circuits linked to epithelial–mesenchymal transition and inflammatory gene expression. These mechanisms intensify pre-existing oncogenic signaling without initiating malignant transformation. Tissue-specific responses are further shaped by IFN competence, renin–angiotensin system balance, and metabolic context. In parallel, immune evasion programs shared by chronic viral infection and cancer, including checkpoint upregulation, impaired antigen presentation, and suppressive myeloid expansion, may be transiently reinforced following severe infection. In contrast, SARS-CoV-2 vaccination induces spatially restricted, self-limited innate activation without sustained inflammatory signaling or persistent antigen exposure. By preventing severe disease and chronic immune dysregulation, vaccination interrupts pathways hypothesized to intersect with cancer biology, with no evidence of increased cancer incidence. Ongoing longitudinal studies are required to clarify the long-term oncologic implications of post-infectious immune remodeling.

## 1. Introduction

The emergence of Coronavirus Disease 2019 (COVID-19), caused by the Severe Acute Respiratory Syndrome Coronavirus 2 (SARS-CoV-2), has presented a global health challenge with far-reaching consequences on public health. Beyond its immediate acute respiratory manifestations, a growing body of evidence indicates that SARS-CoV-2 infection can lead to long-term immunological sequelae, fundamentally altering host immune responses, exacerbating pre-existing conditions or contributing to novel pathologies [1]. The interplay between viral infection, immune evasion, and carcinogenesis is well established in oncology. Chronic viral infections have long been recognized as significant etiological drivers of multiple malignancies, establishing a precedent for evaluating SARS-CoV-2 within a cancer-relevant biological framework [2]. Mechanistically, persistent viral infections promote sustained inflammation, immune dysregulation, oxidative stress, and impaired immune surveillance, thereby creating a biological environment permissive to malignant transformation [3]. Similar processes have been documented in COVID-19, where prolonged cytokine production, oxidative stress, metabolic reprogramming, and T-cell exhaustion may collectively contribute to a pro-tumorigenic milieu [4]. Given these overlaps, an important question emerges as to whether SARS-CoV-2 functions as a direct oncogenic agent or instead represents a transient infection that induces cancer-relevant immune and inflammatory reprogramming in susceptible individuals. In this review, we trace the immunological trajectory of SARS-CoV-2 infection from early interferon imbalance to chronic inflammatory remodeling, examine its convergence with tumor-associated signaling networks, analyze tissue-specific tumor interactions, and contrast these effects with vaccination.

## 2. Acute and Chronic Immune Responses to SARS-CoV-2

**Figure 1:**
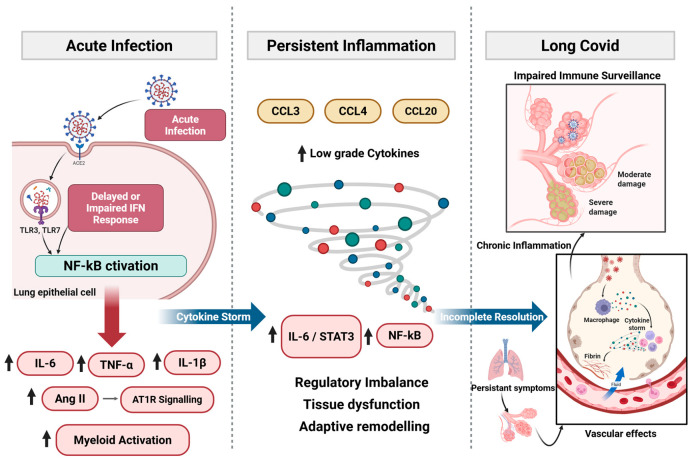
Immune trajectory from acute SARS-CoV-2 infection to chronic immune remodeling. Acute SARS-CoV-2 infection triggers antiviral responses shaped by the balance between early type I/III IFN signaling and NF-κB-driven inflammation. In mild disease, timely IFN responses promote immune resolution. In severe infection, delayed or impaired IFN responses permit sustained NF-κB activation, amplification of IL-6, TNF-α, and IL-1β, Ang II–AT1R signaling, and myeloid activation, contributing to cytokine storm and JAK/STAT3 engagement. Incomplete resolution may lead to persistent low-grade cytokine and chemokine activity, regulatory imbalance, and tissue dysfunction. Long COVID reflects downstream chronic inflammation, impaired immune surveillance, vascular effects, and adaptive immune remodeling. Created with BioRender.com.

SARS-CoV-2 induces immune responses ranging from effective antiviral control to profound dysregulation [5]. The timing and magnitude of innate and adaptive activation determine whether immune homeostasis is restored or whether inflammatory, metabolic, and exhaustion programs persist. These divergent trajectories underline chronic pathology, impaired tissue repair, and altered immune surveillance with potential oncogenic relevance (Figure 1).

### 2.1. Acute Immune Activation and Cytokine Storm

SARS-CoV-2 is recognized by Pattern-Recognition Receptors (PRRs) in airway epithelial cells, macrophages, and dendritic cells, inducing type I and III interferons (IFNs) alongside early pro-inflammatory mediators [6]. In mild or asymptomatic infection, timely IFN signaling restricts viral replication and promotes coordinated adaptive immunity [5]. In severe COVID-19, however, type I/III IFN responses are often delayed or attenuated, impairing early viral control and prolonging innate immune activation. Sustained PRR engagement amplifies Nuclear Factor-kappa B (NF-κB)-dependent signaling, increasing production of interleukin (IL)-6, IL-1β, Tumor Necrosis Factor (TNF)-α, and related mediators [7]. Beyond antiviral effects, type I IFNs normally constrain myeloid activation and regulate cytokine balance. Their insufficiency shifts immunity toward NF-κB-dominant inflammatory amplification. Additional pathways reinforce this inflammatory state. SARS-CoV-2-mediated downregulation of Angiotensin-Converting Enzyme (ACE) 2 enhances Angiotensin (Ang) II–Angiotensin type 1 receptor (AT1R) signaling and Metalloprotease 17 (ADAM17) activation, promoting TNF-α release and generation of soluble IL-6 receptor α [8]. IL-6 trans-signaling via glycoprotein (gp)130–Janus Kinase (JAK)–Signal Transducers and Activators of Transcription (STAT) 3 establishes positive feedback with NF-κB, sustaining inflammation even as viral replication declines [9,10]. Viral RNA and spike protein may further activate Toll-like Receptor (TLR) 2/Myeloid differentiation primary response 88 (MyD88) pathways, augmenting NF-κB-driven cytokine production [11,12]. Clinically, convergence of impaired IFN responses and amplified inflammatory signaling culminates in cytokine release syndrome (CRS), characterized by elevated IL-6, TNF-α, IL-1β, Granulocyte Macrophage Colony-Stimulating Factor (GM-CSF), and chemokines [13]. The pathological significance depends less on peak cytokine levels than on persistence of self-reinforcing NF-κB/JAK–STAT signaling that promotes endothelial activation, vascular leak, and Acute Respiratory Distress Syndrome (ARDS) [8,14].

### 2.2. Transition from Acute to Chronic Immune Dysregulation

As outlined in Section 2.1, early IFN imbalance establishes an NF-κB-dominant inflammatory trajectory that can impair timely resolution, thereby predisposing a subset of individuals to persistent immune dysregulation and chronic inflammatory remodeling [1,14,15,16]. In most patients, contraction of effector responses restores immune equilibrium following viral clearance. However, when acute infection is marked by sustained NF-κB activation, IL-6–STAT3 engagement, and prolonged myeloid stimulation, inflammatory programs may fail to fully resolve. A post-acute immune state can emerge through three partially overlapping mechanisms: (i) residual inflammatory signaling, (ii) persistent perturbation of regulatory pathways, and (iii) adaptive immune remodeling.

Residual inflammation is reflected by continued expression of chemokines such as Macrophage Inflammatory Protein (MIP)-1α (CCL3), Macrophage Inflammatory Protein (MIP)-1β (CCL4), and Macrophage Inflammatory Protein (MIP)-3 (CCL20), indicating ongoing immune cell trafficking and tissue surveillance rather than autonomous inflammatory escalation [17,18]. More consequential is sustained low-grade activation of NF-κB- and STAT3-associated transcriptional programs. Even as circulating cytokines decline, persistent engagement of these pathways may maintain a pro-inflammatory milieu and influence stromal and endothelial function. Prolonged antigen exposure and inflammatory signaling can further drive T-cell exhaustion, altered memory formation, and progressive reduction in naïve lymphocyte pools [15,19]. Thus, the post-acute immune landscape reflects structural reorganization of regulatory circuits rather than simple persistence of acute-phase cytokines. Functionally, this transition manifests low-grade inflammation, altered lymphocyte composition, and impaired immune resolution capacity. The biological impact derives from the stability of dysregulated signaling networks that modulate tissue homeostasis and immune surveillance.

### 2.3. Persistent Inflammation and Immune Remodeling in Long COVID

Long COVID represents a remodeled immune state in which inflammatory resolution remains incomplete after acute infection. Multiple cohorts demonstrate persistent low-grade elevation of cytokines and chemokines, including IL-6, IL-1β, TNF-α, Monocyte Chemoattractant Protein (MCP)-1 (CCL2), Interferon Gamma-Induced Protein (IP)-10 (CXCL10), and CCL4, up to 6–12 months post-infection [20]. Many of these mediators likely reflect continued immune surveillance or delayed tissue repair rather than autonomous cytokine amplification. More biologically significant is sustained low-level NF-κB and JAK/STAT activity despite normalization of systemic cytokine levels. Persistent IL-6-associated signaling and checkpoint ligand expression in subsets of patients suggest incomplete restoration of immune equilibrium [17,21]. This regulatory imbalance may influence endothelial activation, stromal remodeling, and metabolic adaptation. Cellular analyses reveal reduced naïve T- and B-cell pools, expansion of activated monocytes, and enrichment of exhausted or terminally differentiated T-cell subsets months after infection [19,22]. In some phenotypes, including Myalgic Encephalomyelitis/Chronic Fatigue Syndrome (ME/CFS)-like presentations, elevated Galectin-9, Artemin, and autoantibodies indicate convergence of immune activation and exhaustion [23]. Clinically, persistent inflammation correlates with fatigue, cognitive dysfunction, dyspnea, dysautonomia, and chronic pain [24]. Proposed mechanisms include neuroinflammation, microvascular injury, and immunothrombosis [25]. Even when circulating cytokines normalize, extracellular vesicle-associated inflammatory mediators and neuronal injury markers such as Neurofilament Light chain (NfL) and Glial Fibrillary Acidic Protein (GFAP) may remain detectable [26,27], supporting compartmentalized immune activity. Taken together, long COVID reflects sustained regulatory perturbation and adaptive remodeling rather than continuation of acute cytokine storm.

### 2.4. T-Cell Dysfunction, Exhaustion and Impaired Immune Surveillance

Among the most consequential features of post-acute immune remodeling is persistent T-cell dysfunction. During acute severe disease, lymphopenia affects both CD4^+^ and CD8^+^ subsets and correlates with mortality [26,28]. This depletion likely reflects inflammatory redistribution, apoptosis, metabolic stress, and sustained antigen exposure. Increased expression of inhibitory receptors, including Programmed Cell Death (PD)-1, T Cell Immunoglobulin Mucin (TIM-3), Lymphocyte Activation Gene (LAG)-3, and Cytotoxic T-Lymphocyte-Associated (CTLA) protein-4, is common [29,30]. However, elevated inhibitory receptor expression during acute infection may represent transient activation rather than bona fide exhaustion. Consistent with this interpretation, many T cells maintain proliferative potential and cytokine-producing capacity [29,31]. Prolonged inflammatory signaling and persistent antigen exposure may instead drive bona fide exhaustion in susceptible individuals. Exhaustion is characterized by impaired proliferation, reduced effector cytokine production, stable inhibitory receptor expression, transcriptional and epigenetic reprogramming, and metabolic dysfunction [29,32]. Severe and some long-COVID cohorts demonstrate expansion of CD28^−^CD57^+^ or Terminally Differentiated Effector Memory (TEMRA) CD8^+^ populations with reduced regenerative capacity [26,33]. Longitudinal studies indicate persistence of reduced naïve T-cell pools and exhausted or senescent CD8^+^ subsets beyond the acute phase [15,19,34,35,36,37]. Circulating markers of activation and exhaustion correlate with fatigue and cognitive dysfunction [38], suggesting functional relevance. These adaptive alterations may compromise immune surveillance. Persistent inhibitory receptor-high and poorly regenerative T-cell compartments could attenuate the capacity to detect and eliminate transformed cells, particularly in predisposed or immunologically vulnerable hosts. These immune alterations establish the systemic context in which tumor-associated effects must be interpreted.

## 3. Convergence on Tumor-Permissive Signaling Networks

Beyond systemic immune remodeling, SARS-CoV-2 infection engages intracellular signaling pathways that regulate inflammation, proliferation, survival, angiogenesis, and immune evasion, core processes in tumor biology. Rather than initiating de novo transformation, SARS-CoV-2 appears to modulate and intensify pre-existing oncogenic signaling circuits, particularly those involving NF-κB, Janus Kinase (JAK)/Signal Transducers and Activators of Transcription (STAT) 3, Mitogen-Activated Protein Kinase (MAPK)/Extracellular Signal-Regulated Protein Kinases (ERK), and TLR2–MyD88 pathways.

### 3.1. Viral Protein-Mediated Engagement of Host Signaling

SARS-CoV-2 structural proteins can activate inflammatory and stress-response pathways independent of productive replication. The spike (S1) subunit functions as a Pathogen-Associated Molecular Pattern (PAMP)-like ligand that engages NF-κB and MAPK/ERK signaling in epithelial, endothelial, and myeloid cells [11,39,40,41,42], promoting ERK1/2 and p38 phosphorylation, Activator Protein (AP-1)-dependent transcription, and production of IL-6, IL-8, and TNF-α. In macrophages and epithelial cells, spike additionally signals through TLR2/TLR4–MyD88, amplifying NF-κB activation and inflammasome responses [11,43]. The membrane (M) protein similarly modulates oncogenic signaling. In breast cancer models, M expression activates NF-κB and downstream STAT3 signaling, inducing Epithelial–Mesenchymal Transition (EMT) transcription factors and pro-migratory gene programs [44]. NF-κB inhibition attenuates these effects, positioning it upstream of JAK/STAT3 [45]. Although largely derived from in vitro systems, these data suggest that viral proteins potentiate inflammatory and EMT-associated pathways central to tumor progression.

### 3.2. NF-κB–STAT3 Feedback and Inflammatory Stabilization

NF-κB acts as a central hub linking viral sensing to tumor-associated inflammatory programs. Multiple SARS-CoV-2 proteins, including spike, Open Reading Frame (ORF) 3a, and Non-Structural Protein (NSP) 6, activate canonical NF-κB signaling via Transforming Growth Factor (TGF)-β-activated kinase (TAK) 1– Iκ B kinase (IKK) complexes [46,47,48], inducing IL-6 and other mediators involved in proliferation, angiogenesis, and EMT. IL-6-mediated JAK/STAT3 activation establishes a reinforcing feedback loop in which NF-κB-driven cytokine production sustains STAT3 signaling, and STAT3 stabilizes inflammatory and survival gene expression. This reciprocal NF-κB–STAT3 axis is characteristic of tumor-promoting microenvironments and may be engaged during severe or persistent infection. TLR2 serves as an upstream trigger. Upon engagement by spike and envelope proteins, it initiates MyD88-dependent NF-κB activation, thereby driving downstream IL-6/STAT3 signaling [12]. Thus, antiviral sensing pathways can intersect with and transiently reinforce tumor-associated inflammatory circuits.

### 3.3. MAPK/ERK as an Amplification Node

SARS-CoV-2 infection robustly engages the MAPK network, particularly ERK1/2 and p38 signaling pathways [45,49,50]. Early after infection, rapid activation of the Raf–MEK-ERK cascade occurs, and pharmacologic MEK inhibition attenuates both viral replication and cytokine production [51]. Spike-dependent Epidermal Growth Factor (EGFR)–C-RAF–MEK–ERK signaling further links viral sensing to proliferative and survival pathways [52]. In parallel, p38 activation amplifies inflammatory cytokine production. Pharmacologic inhibition attenuates IL-6, IL-8, and CXCL10 levels while preserving interferon responses [53]. Sustained MAPK activity is reinforced by suppression of regulatory phosphatases such as Dual-Specificity Protein Phosphatase (DUSP)1 and DUSP5, thereby prolonging ERK, p38, and NF-κB signaling [45,54]. Given that ERK regulates MYC expression, cell-cycle progression, and survival programs, and that p38 contributes to angiogenesis, fibrosis, and metastatic adaptation, infection-induced MAPK activation represents a mechanistic bridge between inflammatory stress and tumor-permissive microenvironments. These findings indicate pathway reinforcement within tumor-relevant signaling networks.

### 3.4. Integrated Network Perspective

The mechanisms outlined above reveal a tightly interconnected signaling architecture in which NF-κB, JAK/STAT3, and MAPK/ERK function as regulatory hubs integrating antiviral sensing with programs governing survival, angiogenesis, EMT, and immune checkpoint expression. When early IFN responses are blunted and NF-κB-dominant inflammation persists, this network may remain transiently reinforced, particularly in tissues already primed by oncogenic or inflammatory stress [46,49,50]. In this context, viral protein-mediated signaling can augment existing proliferative and survival programs without conferring autonomous transforming capacity.

The emergence of variants with altered transmissibility and immune evasion properties adds further complexity to the infection–cancer interface. Omicron sub-lineages, for example, exhibit differences in IFN antagonism, spike–receptor interactions, and inflammatory signatures that may influence the magnitude and duration of pathway engagement [55]. Whether such variant-specific differences translate into differential modulation of tumor-relevant signaling remains largely unknown. Comparative mechanistic analyses across variants therefore represent an important research direction.

## 4. Tumor Microenvironment and Tissue-Specific Cancer Responses

While SARS-CoV-2 is not a transforming virus, its driven immune remodeling and protein-mediated signaling can intersect with tumor microenvironments in tissue-specific ways. Tumors exhibit altered receptor expression, impaired antiviral signaling, metabolic stress, and pre-existing inflammatory niches, conditions that may modulate susceptibility to viral exposure and downstream signaling effects.

### 4.1. Direct or Abortive Infection of Cancer Cells

Experimental evidence indicates that selected cancer cell types can support SARS-CoV-2 entry and, in some contexts, productive or abortive infection. Hepatoma cell lines such as Huh7.5 and HepG2 permit viral replication with associated cytopathic effects, whereas non-transformed hepatic progenitors remain largely non-permissive, suggesting that malignant transformation and altered innate immune signaling influence cellular susceptibility [56]. Serial passages in hepatoma models promote selection of spike-adapted variants with enhanced replication efficiency and reduced reliance on canonical ACE2-mediated entry [38,56]. In glioblastoma models, permissiveness correlates with ACE2 expression and impaired IFN responses, and ACE2 overexpression further increases susceptibility [57]. In addition to ACE2, alternative receptors, including Asialoglycoprotein receptor (ASGR) 1, Dendritic Cell-Specific Intercellular adhesion molecule-3-Grabbing Non-integrin (DC-SIGN), Transmembrane protein (TMEM) 106B, and heparan sulfate-associated mechanisms, may facilitate viral entry within tumor-associated cellular contexts [55,58]. Importantly, viral exposure can induce phenotypic remodeling even in the absence of sustained replication. In hepatic progenitor models, spike exposure promotes partial dedifferentiation and syncytia formation despite limited viral propagation [56]. These findings suggest that tumor cells may undergo transient infection or signaling perturbation; however, there is no evidence for viral genome integration or direct oncogenic transformation.

### 4.2. Tumor Microenvironment Remodeling and Checkpoint Induction

In vivo studies and spatial transcriptomic analyses indicate that tumor tissues may harbor viral RNA or exhibit virus-associated response signatures following SARS-CoV-2 infection [59]. Within virus-enriched tumor regions, increased Programmed Cell Death Ligand (PD-L) 1 expression, features of T-cell dysfunction, and B-cell-rich immune niches have been reported, consistent with localized remodeling of immune architecture. In this setting, checkpoint upregulation most likely reflects cytokine-driven inflammatory signaling rather than viral transformation. Type I and II IFNs, NF-κB activation, and IL-6–STAT3 signaling are established inducers of PD-L1 transcription, and sustained cytokine exposure can reinforce adaptive immune resistance. Accordingly, tumor cell “remodeling” appears to represent inflammation-mediated phenotypic adaptation under persistent immune pressure. Prolonged exposure to IL-6, TNF-α, and related mediators may further promote EMT, angiogenesis, stromal activation, and metabolic reprogramming within the tumor microenvironment [56,59]. These processes overlap with the signaling networks described in Section 3 and could be amplified in tissues subjected to sustained inflammatory stress.

### 4.3. Tissue-Specific Divergence in Cancer Responses

SARS-CoV-2-associated effects vary substantially across tumor types. Divergent responses appear to be influenced by ACE2 and Transmembrane Protease, Serine (TMPRSS) 2 expression patterns, IFN competence, renin–angiotensin–aldosterone system (RAAS) signaling balance, and metabolic context [60]. In colorectal cancer models, where ACE2 and TMPRSS2 expressions are relatively high, viral exposure has been linked to enhanced inflammatory and pro-migratory signaling [61,62]. In contrast, prostate cancer models exhibit context-dependent susceptibility, potentially shaped by androgen-regulated TMPRSS2 expression and differences in interferon responsiveness [63,64]. Downregulation of ACE2 in ACE2-high gastrointestinal and lung tumors may amplify Ang II–AT1R signaling, thereby increasing Reactive Oxygen Species (ROS) production, hypoxia, angiogenesis, and inflammatory cytokine release. Conversely, baseline ACE2 expression in certain tumor contexts has been associated with reduced stemness and more favorable immune signatures, highlighting that ACE2 biology may exert tumor type-specific effects independent of viral infection [65,66]. IFN competence further modulates these outcomes. Tumors with impaired interferon signaling may permit sustained viral pathway engagement and inflammatory amplification, whereas interferon-high tumors preferentially induce truncated deltaACE2 isoforms that lack spike-binding capacity, thereby limiting additional viral entry [67,68].

### 4.4. Inflammation-Driven Reactivation of Dormant Metastases

Tissue-specific inflammatory remodeling may also influence metastatic dormancy. In murine breast cancer models, SARS-CoV-2-induced lung inflammation disrupted dormancy programs in disseminated tumor cells, triggering IL-6-dependent metastatic outgrowth without direct viral infection of tumor cells [69]. Infection-associated impairment of T-cell activation and altered CD4^+^/CD8^+^ dynamics contributed to sustained metastatic burden. Observational analyses in cancer survivors have suggested increased lung metastasis and cancer-related mortality following SARS-CoV-2 infection [69], although causality remains to be established. These findings emphasize that infection-induced tissue inflammation, rather than direct viral oncogenesis, may under specific conditions facilitate tumor progression. Across tumor contexts, SARS-CoV-2-associated effects appear to arise primarily from immune and inflammatory remodeling rather than direct genetic transformation. The magnitude and direction of these effects depend on tumor-intrinsic signaling programs, antiviral competence, RAAS balance, and the local immune milieu. Together, these observations support a model in which SARS-CoV-2 acts as a context-dependent modifier of tumor ecosystems, with consequences shaped by pre-existing vulnerabilities rather than uniform pro-oncogenic activity.

## 5. Immune Evasion Architecture Shared by SARS-CoV-2 and Cancer

**Figure 2:**
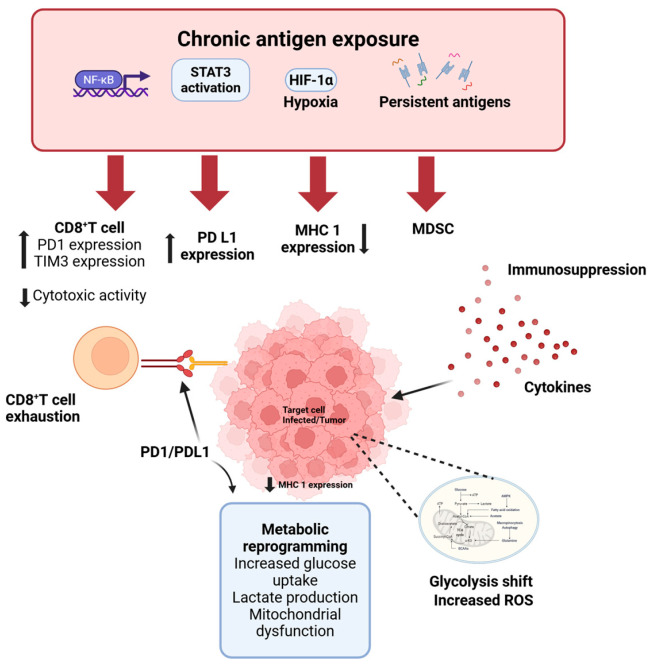
Shared Immune evasion mechanisms in SARS-CoV-2 infection and cancer. Chronic antigen exposure in severe or persistent SARS-CoV-2 infection, similar to cancer, promotes sustained activation of NF-κB, STAT3, and HIF-1α pathways under inflammatory and hypoxic conditions. This environment drives upregulation of PD-L1, expansion of myeloid-derived suppressor cells (MDSCs), and reduced MHC class I expression on target cells. CD8^+^ T cells progressively acquire an exhausted phenotype characterized by increased PD-1 and TIM-3 expression and diminished cytotoxic activity. Concurrent metabolic reprogramming further reinforces immunosuppression. These convergent mechanisms impair immune surveillance and promote persistence of infected or malignant cells. Created with BioRender.com.

While Section 3 and Section 4 address signaling convergence and tumor-context interactions, an equally important dimension concerns immune surveillance. Both cancer and SARS-CoV-2 infection employ mechanisms that limit antigen visibility, induce checkpoint pathways, and reshape the myeloid compartment, thereby weakening cytotoxic immune control (Figure 2).

### 5.1. Immune Checkpoint Upregulation and T Cell Exhaustion Programs

Both chronic SARS-CoV-2 infection and cancer induce inhibitory immune checkpoints that constrain CD8^+^ and CD4^+^ T-cell function. Severe COVID-19 and long COVID are characterized by elevated PD-1, TIM-3, LAG-3, and T-cell immunoglobulin and immunoreceptor tyrosine-based inhibitory Motif (ITIM) structural domain (TIGIT) expression on T cells, often accompanied by reduced proliferation and cytokine production [29,70]. These phenotypes resemble exhaustion programs observed in tumor-infiltrating lymphocytes. Mechanistically, checkpoint induction reflects sustained inflammatory signaling and hypoxic stress. Type I/II IFNs, NF-κB activation, and IL-6–STAT3 signaling can promote PD-L1 expression in epithelial and tumor cells, while Hypoxia-Inducible Factor (HIF)-1α and TGF-β pathways further reinforce immunosuppressive transcriptional programs [70]. In cancer, similar circuits establish adaptive immune resistance and therapeutic escape. The convergence of chronic antigen exposure and inflammatory signaling thus generates parallel exhaustion landscapes in viral infection and malignancy. Importantly, SARS-CoV-2 does not initiate these programs de novo but transiently amplifies pathways already central to tumor immune evasion.

### 5.2. Suppression of Antigen Presentation and Microenvironmental Reprogramming

Downregulation of antigen presentation represents a shared axis of immune evasion. SARS-CoV-2 variants impair Major Histocompatibility Complex (MHC) class I surface expression through coordinated viral protein activity and suppression of NOD-Like Receptor family CARD domain containing (NLRC) 5-dependent transcription, thereby reducing CD8^+^ T-cell recognition [71,72,73,74,75]. Cancer cells employ analogous mechanisms, including genetic loss, epigenetic silencing, and oncogenic MAPK or MYC signaling, to diminish antigen visibility and promote resistance to immune surveillance and immunotherapy. Both SARS-CoV-2 infection and cancer also foster immunosuppressive microenvironments enriched in Myeloid-Derived Suppressor Cells (MDSCs) and regulatory T cells [71,72]. COVID-19-associated myeloid populations exhibit metabolic reprogramming, arginase activity, ROS production, and PD-L1 expression resembling tumor-associated MDSCs, collectively restraining CD8^+^ and NK cell function. Metabolic remodeling further stabilizes this suppressive state. Infected cells shift toward glycolysis, HIF-1α stabilization, mitochondrial dysfunction, and ROS accumulation, adaptations that parallel the Warburg-like phenotype of tumors [76,77]. Beyond supporting viral replication, these changes create metabolically restrictive conditions that compromise cytotoxic lymphocyte responses. Together, impaired antigen presentation, checkpoint induction, myeloid expansion, and metabolic adaptation form an integrated immune evasion architecture common to both SARS-CoV-2 infection and cancer. Although typically transient, these programs may persist in severe or unresolved infections, particularly in immunocompromised hosts. In this context, SARS-CoV-2 does not initiate malignant transformation but can transiently reinforce tumor-associated immune escape networks through inflammatory and metabolic reprogramming.

## 6. SARS-CoV-2 Vaccination as a Protective Modifier of Cancer-Relevant Immune Pathways

### 6.1. Mechanistic Contrast: Infection vs. Vaccination

SARS-CoV-2 vaccination provides a mechanistic contrast to natural infection with respect to antigen persistence, tissue dissemination, and engagement of inflammatory signaling networks. Natural infection involves replicating viruses and sustaining antigen production across multiple tissue compartments. In contrast, currently deployed mRNA and adenoviral vector vaccines encode spike alone and result in transient antigen expression primarily within professional antigen-presenting cells, without viral replication [69,78]. This distinction has important implications for immune remodeling. Whereas infection can sustain NF-κB, MAPK, and IL-6–STAT3 activation in epithelial, endothelial, and myeloid compartments, vaccine-induced innate activation is acute and self-limited, resolving as antigen is cleared. Clinical and immunogenicity studies in patients with solid and hematologic malignancies demonstrate effective spike-specific priming without persistent antigenemia or chronic inflammatory activation [69,78,79,80,81,82]. Adaptive responses also differ qualitatively. Prolonged antigen exposure during severe infection, particularly in immunocompromised hosts, is associated with lymphopenia and accumulation of inhibitory receptor-expressing T cells with exhaustion-like features [83,84,85]. Vaccination induces polyfunctional, Th1-skewed CD4^+^ and CD8^+^ T-cell responses targeting multiple spike epitopes [85,86,87], without establishing sustained exhaustion programs. Recent advances in RNA delivery platforms further refine this distinction. Extracellular vesicle (EV)-based mRNA therapeutics represent an emerging strategy aimed at enhancing targeted antigen delivery while minimizing systemic inflammatory exposure. EV-based systems may improve cellular specificity and reduce off-target distribution, thereby further restricting spike expression to professional APCs and limiting unintended tissue exposure [88]. Such approaches reinforce the principle that vaccine-induced antigen presentation is transient, spatially controlled, and mechanistically distinct from the widespread viral replication observed during infection.

In addition to vaccination, emerging antiviral strategies may further limit infection-driven immune remodeling. Small interfering RNA (siRNA)-based therapeutics targeting conserved regions of the SARS-CoV-2 genome represent a promising approach to suppress viral replication and accelerate viral clearance [89]. By directly reducing viral RNA levels, such strategies may prevent prolonged antigen exposure and downstream NF-κB/IL-6–STAT3 amplification, thereby mitigating chronic inflammatory circuits implicated in tumor-permissive immune states. Although still investigational, siRNA-based antivirals conceptually reinforce the importance of early viral control in preventing the transition from acute interferon imbalance to chronic NF-κB-dominant inflammation.

### 6.2. Impact on Cancer Patients

In patients with solid tumors and hematologic malignancies, vaccine-elicited cellular immune responses are often preserved even when humoral responses are attenuated by chemotherapy or B-cell-depleting therapies. Booster doses enhance both antibody and T-cell responses and are generally well tolerated in oncology populations [79,80,82,90]. Vaccine responsiveness varies across oncologic subgroups. Patients receiving B-cell-depleting agents, such as anti-CD20 monoclonal antibodies, frequently demonstrate impaired seroconversion, with protection relying predominantly on cellular immunity. The durability of T-cell-mediated protection in these settings remains incompletely defined, and optimal booster timing relative to immunosuppressive therapy continues to be actively investigated. These considerations highlight the need for individualized vaccination strategies and longitudinal immune monitoring in highly immunocompromised patients. Importantly, vaccination substantially reduces severe COVID-19, hospitalization, and mortality in cancer populations [78,81,82,91]. By preventing severe infection, vaccination mitigates the prolonged inflammatory signaling, immune dysregulation, and treatment disruption associated with SARS-CoV-2-driven immune remodeling. In this context, infection prevention itself represents a key strategy for limiting cancer-relevant immune perturbations.

### 6.3. Interaction with Immunotherapy

Mechanistic data suggest that transient vaccine-induced interferon signaling may, in certain contexts, intersect positively with antitumor immunity. Preclinical models demonstrate enhanced dendritic-cell priming and epitope spreading against tumor-associated antigens following mRNA vaccination [86]. Retrospective analyses have reported improved outcomes in subsets of patients with non-small-cell lung cancer and melanoma receiving immune checkpoint blockade around the time of vaccination, although these findings remain associative. Importantly, studies in patients receiving PD-1/PD-L1 inhibitors show preserved vaccine immunogenicity without increased immune-related adverse events [85,91,92]. These observations suggest that vaccine-induced immune activation is compatible with, and may occasionally synergize with, immune checkpoint therapy rather than exacerbating immune toxicity.

### 6.4. Epidemiologic Safety

Speculative concerns regarding long-term oncologic effects of repeated vaccination lack empirical support. Extensive pharmacovigilance and population-level analyses have not demonstrated increased cancer incidence following vaccination. Current evidence does not support vaccine-associated tumorigenesis, and such proposals remain hypothesis-generating rather than causal [93]. Natural infection and vaccination occupy opposite ends of the immune remodeling spectrum. Infection may impose prolonged antigen exposure, sustained inflammatory signaling, and adaptive exhaustion in susceptible individuals. Vaccination delivers a transient, spatially restricted antigen pulse that supports durable adaptive immunity without establishing persistent inflammatory niches. These mechanistic distinctions are essential when interpreting post-COVID immune alterations in cancer biology and reinforce the safety and clinical benefit of vaccination in oncology settings.

## 7. Clinical Implications

Cancer patients with SARS-CoV-2 infection experience disproportionately severe outcomes, reflecting baseline immune compromise, systemic inflammation, and vulnerability to treatment disruption. Across large registries, reported mortality rates have ranged from 17–33%, particularly among individuals with hematologic malignancies, advanced age, comorbidities, or recent cytotoxic or monoclonal antibody therapy [93,94]. Profound lymphopenia and elevated inflammatory markers—including IL-6, CRP, LDH, and neutrophil-to-lymphocyte ratio—predict both acute severity and prolonged recovery [95,96,97,98]. Beyond acute mortality, infection frequently disrupts oncologic care. Treatment delays of several weeks are common and may adversely affect tumor control in selected high-risk settings [99]. Persistent inflammatory signatures and post-acute sequelae raise concerns regarding sustained immune dysregulation in cancer survivors, although definitive evidence linking SARS-CoV-2 infection to accelerated tumor progression remains limited. Careful follow-up and risk stratification may therefore be warranted in vulnerable populations. Preventive strategies substantially mitigate these risks. In oncology populations, vaccination reduces severe disease, hospitalization, and mortality, thereby limiting infection-associated inflammatory remodeling and preserving treatment continuity [78,81,82,91]. Available data demonstrates preserved immunogenicity in many patients with solid tumors and acceptable safety profiles, including among those receiving immune checkpoint inhibitors. To date, no evidence supports increased cancer incidence following vaccination. The COVID–cancer interface thus underscores both vulnerability and opportunity: heightened susceptibility to infection necessitates proactive prevention, while mechanistic insights into inflammatory and immune regulatory networks inform risk stratification and therapeutic decision-making. Maintaining infection control and minimizing prolonged inflammatory stress are central to preserving oncologic outcomes.

## 8. Conclusions and Future Directions

SARS-CoV-2 does not fulfill the criteria of a classical oncogenic virus. It lacks dedicated transforming oncogenes, does not integrate into the host genome, and does not establish stable latency comparable to established oncoviruses such as HPV, HBV, or EBV [84,100]. Current evidence therefore does not support direct viral oncogenesis [66]. Nevertheless, SARS-CoV-2 infection perturbs immune and intracellular signaling networks that substantially overlap with tumor-promoting pathways [92]. Early interferon imbalance followed by sustained inflammatory amplification, centered on NF-κB, IL-6/JAK–STAT3, and MAPK signaling, may, under susceptible conditions, transiently reinforce tumor-supportive microenvironments [4,53]. Severe infections and prolonged post-acute syndromes are characterized by immune exhaustion, impaired cytotoxic surveillance, oxidative stress, fibrosis, and tissue remodeling, features that resemble microenvironmental hallmarks of cancer progression rather than viral transformation [101]. These observations support a model in which SARS-CoV-2 modulates pre-existing signaling circuits rather than initiating malignancy [66]. The biological impact likely depends on the magnitude and duration of inflammatory remodeling, host immune competence, tumor-intrinsic vulnerabilities, and tissue context. Vaccination provides a critical counterbalance. By delivering a transient, spatially restricted antigen exposure without sustained viral replication, vaccination prevents the prolonged inflammatory states implicated in tumor-relevant immune remodeling [53,92]. In doing so, it interrupts the cascade linking acute interferon imbalance to chronic NF-κB-dominant inflammation and immune dysfunction. Important research gaps remain. These include variant-specific differences in immune remodeling, the long-term oncologic consequences of post-acute inflammatory states, and the durability of protective immunity in profoundly immunosuppressed populations [102]. Addressing these questions will require longitudinal epidemiologic surveillance, mechanistic studies of persistent immune alterations, and biomarker-guided risk stratification in cancer survivors [102]. The appropriate stance is neither undue alarm nor premature reassurance, but rigorous, evidence-based vigilance [53,92]. Within this framework, SARS-CoV-2 is best understood not as an oncogenic virus, but as a transient immunological stressor whose influence on cancer biology is contingent on host vulnerability, inflammatory persistence, and effective preventive intervention.

## Data Availability

No new data were created or analyzed in this study. Data sharing is not applicable to this article.
